# Small Prokaryotic Proteins Interacting with Nucleic Acids

**DOI:** 10.3390/ijms23179876

**Published:** 2022-08-30

**Authors:** Alicja Węgrzyn, Grzegorz Węgrzyn

**Affiliations:** 1 Laboratory of Phage Therapy, Institute of Biochemistry and Biophysics, Polish Academy of Sciences, Kładki 24, 80-822 Gdańsk, Poland; 2 Department of Molecular Biology, Faculty of Biology, University of Gdańsk, Wita Stwosza 59, 80-308 Gdańsk, Poland

Among proteins that interact with DNA or RNA, more or less specifically, there is a special group of relatively small polypeptides which are present in prokaryotic cells and interact with nucleic acids. The roles of these proteins were previously somewhat underestimated, but now it is clear that they have crucial functions in various essential processes in bacteria and archaea, such as DNA replication, genetic recombination, gene expression regulation, and others. The Special Issue of this journal, entitled “Small Prokaryotic Proteins Interacting with Nucleic Acids” has been devoted to these proteins.

A summary of the roles of small prokaryotic proteins in protecting genome integrity in bacterial and archaeal cells, as well as discussions on the importance of such nucleoid-associated proteins, have been presented by Molan and Žgur Bertok [1]. They described our current understanding of various mechanisms by which different small proteins ensure the integrity of DNA in prokaryotes. Specific proteins were discussed in more detail, such as Dps which protects genomes according to two major mechanisms, HU which regulates gene transcription and acts in the signal transduction processes, CbpA which has a co-chaperone function, and SSB proteins reveling properties of binding to single-strand DNA and thus playing a general role in genome maintenance. Interestingly, archaeal cells utilize various strategies to achieve accurate organization and protection of their genomes, some of them being similar to those occurring in eubacteria while others resembling mechanisms found in eukaryotic organisms. The review article by Molan and Žgur Bertok [1] is, therefore, an excellent overview of various mechanisms ensuring proper structures and functions of genomes under different conditions and indicates further ways of research which should allow for understanding these processes in more detail in the future.

A series of four original articles included in this Special Issue provides examples of the huge variability of small prokaryotic proteins interacting with nucleic acids. Huang et al. [2] reported the identification of a previously unknown family of DNA-binding proteins from Archaea. Although the title of their article, in which the authors suggested the discovery of a “novel family of proteins”, might be somewhat misleading (as these proteins existed in nature for millions or even billions of years, and were “only” newly discovered, but not new in the meaning of newly constructed), the demonstrated results are very important. The newly identified family of winged-helix single-stranded DNA binding proteins has been named Sul7s. These proteins are conserved in Sulfolobaceae and reveal strong binding to single-stranded DNA, several times more efficient than to double-stranded DNA. Three α-helices, three β-strands, and two short wings are present in the structures of Sul7s which is characteristic of the winged helix-turn-helix fold. These results made a significant contribution to understanding the variability of single-stranded DNA binding proteins in Archaea and evolutionary relationships between these organisms.

The work by Aseev et al. [3] concerns the regulation of expression of the *rpsO* gene, coding for the S15 protein (one of the ribosomal proteins from the small ribosome subunit) in *Mycobacterium smegmatis* and *M. tuberculosis*. Using novel genetic tools (constructed by Aseev et al. [3]) they found that regulation of the *rpsO* gene expression occurs mainly at the translation stage in *M. smegmatis* and *M. tuberculosis*, and proceeds according to the negative feedback mode. The formation of the pseudoknot in the *rpsO* 5′UTR region appeared to be essential for the S15 protein-mediated repression of translation of its own mRNA. Interestingly, similar regulation of the *rpsO* gene expression was also found in *Escherichia coli*, indicating that the same mechanism of control of the S15 ribosomal protein production occurs in as different prokaryotic organisms as enterobacterial and mycobacterial cells.

The role of the Hfq protein, a small polypeptide able to interact with both RNA and DNA, in a chromosome- and plasmid-borne resistance of *E. coli* to high concentrations of antibiotics has been studied by Gaffke et al. [4]. Since local concentrations of antibiotics after their administration to humans or animals might be temporary many times higher than those occurring during longer periods in their bodies, such studies might have not only basic but also practical meaning. It was found that the activity of Hfq was required for the survival of bacterial cells bearing plasmids with genes coding for proteins causing resistance to standard concentrations of chloramphenicol, tetracycline, or ampicillin at elevated levels of these antibiotics. Intriguingly, the presence of the active form of the Hfq protein resulted in a low level of resistance to high concentrations of kanamycin, ensured by the presence of the chromosomally located *kan* gene. Such resistance patterns to various antibiotics were found to be intrinsic rather than arising from specific adaptation. In bacteria bearing plasmids with genes encoding antibiotic-resistance proteins, the correlation of the resistance to high antibiotic concentrations to the presence of Hfq functions could be observed mainly in the case of a low-copy number plasmid but not in the presence of a medium- or high-copy number plasmid. Studies on the ColE1-like plasmids suggested that Hfq may cooperate with the plasmid-encoded Rom protein (another small polypeptide, known as a factor facilitating RNA-RNA interactions) in the process of the regulation of plasmid replication. Therefore, these studies contributed to our understanding of the roles of some specific small proteins interacting with nucleic acids in as different processes such as development of antibiotic resistance and control of DNA replication.

The structure-function relationships in the bacteriophage λ Beta protein (also known as Redβ), a component of the Red recombination system (one of the most effective recombination systems known among prokaryotic and eukaryotic organisms) have been investigated by Zakharova et al. [5]. A high-resolution crystal structure of this DNA-interacting protein playing a crucial role in the Red recombination is still not available. Only some models of the Beta DNA binding domain were proposed by comparison with the eukaryotic Rad52 protein. The only reported crystal structure of Beta comprises the C-terminal domain, responsible for binding to the Exo protein (the phage λ-encoded exonuclease). Therefore Zakharova et al. [5] performed experiments that facilitated testing the predicted models of the Beta protein by comparing the recombination activities of polypeptides in which various lysine residues were replaced with alanine ones. In this way, critical amino acid residues could be identified which are essential for DNA binding, DNA annealing, and recombination in vivo. The above-mentioned experiments helped to corroborate previously proposed models of the structure of the Beta protein, and provided important data indicating specific roles of particular amino acid residues in the biochemical functions of this polypeptide.

In summary, articles published in the Special Issue of this journal, devoted to small prokaryotic proteins interacting with nucleic acids, provided important new information on structures and functions of various such polypeptides, involved in as different cellular processes as interactions with single-stranded DNA, regulation of gene expression at the stage of translation initiation, control of chromosome- and plasmid-borne resistance to high concentrations of antibiotics, and genetic recombination. Moreover, an overview of the role of such proteins in the protection of genome integrity has been presented. Thus, this Special Issue provides an important contribution to the field, indicating that further studies in this research area are crucial to understand the functions of these extraordinary proteins.
